# Role of the Transcriptional Corepressor *Bcor* in Embryonic Stem Cell Differentiation and Early Embryonic Development

**DOI:** 10.1371/journal.pone.0002814

**Published:** 2008-07-30

**Authors:** Joseph Alan Wamstad, Connie Marie Corcoran, Anne Marjorie Keating, Vivian J. Bardwell

**Affiliations:** 1 Molecular, Cellular, Developmental Biology and Genetics Graduate Program, University of Minnesota, Minneapolis, Minnesota, United States of America; 2 Department of Genetics, Cell Biology and Development and Cancer Center, University of Minnesota, Minneapolis, Minnesota, United States of America; 3 Biochemistry, Molecular Biology and Biophysics Graduate Program, University of Minnesota, Minneapolis, Minnesota, United States of America; City of Hope Medical Center, United States of America

## Abstract

*Bcor* (BCL6 corepressor) is a widely expressed gene that is mutated in patients with X-linked Oculofaciocardiodental (OFCD) syndrome. BCOR regulates gene expression in association with a complex of proteins capable of epigenetic modification of chromatin. These include Polycomb group (PcG) proteins, Skp-Cullin-F-box (SCF) ubiquitin ligase components and a Jumonji C (Jmjc) domain containing histone demethylase. To model OFCD in mice and dissect the role of Bcor in development we have characterized two loss of function *Bcor* alleles. We find that Bcor loss of function results in a strong parent-of-origin effect, most likely indicating a requirement for *Bcor* in extraembryonic development. Using *Bcor* loss of function embryonic stem (ES) cells and in vitro differentiation assays, we demonstrate that *Bcor* plays a role in the regulation of gene expression very early in the differentiation of ES cells into ectoderm, mesoderm and downstream hematopoietic lineages. Normal expression of affected genes (*Oct3/4*, *Nanog*, *Fgf5*, *Bmp4*, *Brachyury* and *Flk1*) is restored upon re-expression of *Bcor*. Consistent with these ES cell results, chimeric animals generated with the same loss of function *Bcor* alleles show a low contribution to B and T cells and erythrocytes and have kinked and shortened tails, consistent with reduced *Brachyury* expression. Together these results suggest that *Bcor* plays a role in differentiation of multiple tissue lineages during early embryonic development.

## Introduction

Mutations causing gene fusions, loss of function or misregulation of transcriptional corepressor proteins have been implicated both in genetically inherited diseases and cancer [1], [2]. BCOR (BCL6 corepressor) is a transcriptional corepressor that was originally identified by its ability to interact with the site specific transcriptional repressor BCL6 [3]. BCOR is found in an 800 kDa complex in which at least two proteins have chromatin modifying activity: the PcG transcriptional repressor protein, RNF2, is a histone H2A E3 ubiquitin ligase and FBXL10 (JHDM1B, KDM2B) is a Jmjc histone demethylase in addition to its presumed ubiquitin E3 ligase activity [4], [5]. BCL6 plays critical roles in specific immunological processes involving B and T cells, including germinal center formation and the generation and maintenance of memory T cells [6]–[10]. In addition, testicular germ cell apoptosis and defects in erythropoiesis have been reported in BCL6-deficient mice [11], [12]. Deregulated expression of BCL6, due to chromosomal translocations or point mutations, is associated with the formation of approximately one sixth of non-Hodgkin's lymphomas [13], [14]. In cell lines derived from such patients, BCOR is detected at a number of BCL6 target genes suggesting that BCOR is likely to play a role in mediating BCL6 driven lymphomagenesis. Since its original discovery, BCOR has been shown to directly interact with the transcriptional regulator AF9 (MLLT3), and to be in a complex with ENL (MLLT1). AF9 and ENL are known MLL (trithorax) fusion partners in acute leukemias [15]. AF9 itself is a regulator of Hox gene expression and skeletal development [16], [17].

In addition, BCOR has been shown to play multiple roles in the complex process of human development. Females who are heterozygous for X-linked *Bcor* mutations have the rare Oculofaciocardiodental (OFCD) syndrome, the primary subtype of OMIM #300166 microphthalmia, syndromic 2 (MCOPS2) [18]. Congenital disorders in patients suffering from OFCD include cataracts, microphthalmia, and cardiac, dental and digital anomalies [18], [19]. In the hematopoietic lineage of OFCD patients, *Bcor* clearly is critical as 90–100% of surviving white blood cells show inactivation of the X- chromosome carrying the mutant allele of *Bcor* [18]. Presumably, the selective disadvantage, caused by an active X-chromosome harboring a *Bcor* mutation, is less severe in some tissues leading to variable phenotypic effects in a mosaic fashion. Male OFCD patients do not exist and are presumed to die in early development. Almost all *Bcor* mutations in OFCD patients result in premature stop codons that are thought to cause nonsense-mediated decay of the mRNA [18]. The second form of MCOPS2, Lenz microphthalmia, results from a single missense mutation (p. P85L) in the fourth coding exon of *Bcor* and is inherited in an X-linked recessive pattern [18]. Patients with Lenz microphthalmia suffer from microphthalmia/anophthalmia, mental retardation, and skeletal and other anomalies [18], [20].

The severity and breadth of these two MCOPS2 syndromes illustrate the important roles *Bcor* plays during development. Currently two animal models have successfully recapitulated developmental abnormalities similar to those found in patients with OFCD and Lenz microphthalmia. RNAi knock down of *Bcor* in zebrafish (*Danio rerio*) results in colobomatous eye defects and perturbations in somite, skeletal and neural tube development [18]. Colobomas, microphthalmia and cardiac abnormalities were also seen in morpholino knock down of *Bcor* in frogs (*Xenopus tropicalis*). Additionally, laterality defects were observed in *Bcor* morpholino injected frogs, similar to those present in a subset of OFCD patients [21].

The expression of *Bcor* during mouse development correlates well with tissues and organs in adversely affected patients with OFCD or Lenz microphthalmia [22]. Three separate promoters differentially control the expression of *Bcor* through embryonic development and into adulthood. Section *in situ* analysis shows that *Bcor* mRNA is expressed strongly in extraembryonic tissues during gastrulation and embryonic turning, suggesting a significant role in placental formation. *Bcor* expression is upregulated in the embryo proper at embryonic day 9 in mice starting in the tail, limb buds and branchial arches. During the later fetal stages of mouse development, *Bcor* is expressed in multiple tissues including strong expression in lens and retina of the eye and the neural tube [22].

The abnormalities present in *Bcor* knockdown fish and frogs, combined with the severe clinical presentation of MCOPS2 syndromes in humans, clearly illustrate the importance of *Bcor* in development. The developmental requirement for *Bcor*, together with its potential role in BCL6-related lymphomagenesis, underscores the importance of generating and analyzing mutant alleles of *Bcor*. To this end, we have analyzed the effect of two independent *Bcor* loss of function alleles on early development in mice and in ES cell differentiation. We found that *Bcor* exhibits a parent-of-origin effect, in that only paternal transmission results in viable offspring. Since the paternal X chromosome is exclusively inactivated in the extraembryonic tissue, this strongly suggests *Bcor* is required for extraembryonic development. In addition, loss of *Bcor* results in compromised development during *in vitro* differentiation of embryonic stem cells.

## Results

### Generation and analysis of a conditional *Bcor* allele

**Figure 1:**
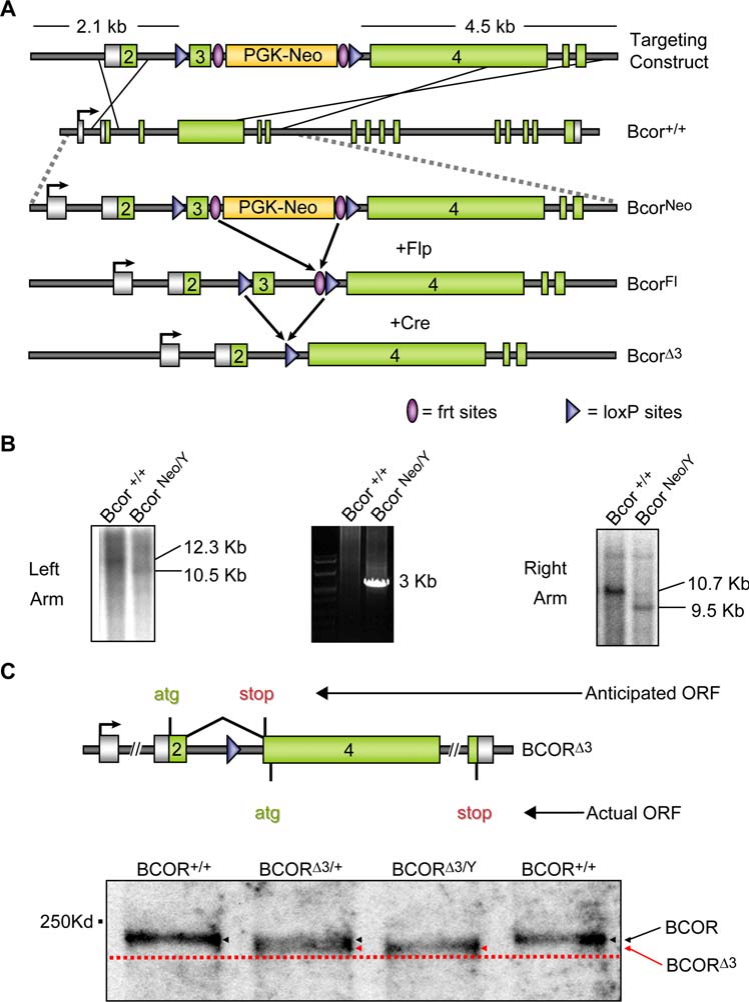
Generating *Bcor* ^Δ^ ^*3*/+^ and *Bcor* ^Δ^ ^*3/Y*^ mice. (A) Diagram of targeting construct and recombination strategy. The *Bcor^+^* allele was targeted by homologous recombination to generate *Bcor^Neo^* embryonic stem cells. This allele contains loxP sites flanking the third exon, and a *Neo* resistance cassette flanked by frt sites, internal to the loxP sites immediately downstream of exon 3. Expression of Flp and/or Cre site specific recombinases *in vivo* or *in vitro* can be used to excise the intervening sequence between their respective recognition sites, frt and loxP, generating *Bcor^Fl^* and *Bcor* ^Δ*3*^ alleles. PGK-Neo (phosphoglycerate kinase promoter–driven neomycin resistance gene cassette) (B) Southern blot and PCR analyses of targeted and wild type ES cell clones. Genomic DNA from individual ES cell clones was digested with the restriction enzymes, *BamH1* (left arm) and *BglII* (right arm). The 5′ external probe (Left Arm) hybridizes to 10.7-kb (*Bcor^+/Y^*) and 9.5-kb (*Bcor^Neo/Y^*) *BamH1* fragments. Given the weak left arm signal, confirmatory PCR was performed on genomic DNA from wild type and targeted ES cells using a forward primer 5′ to the left arm and a reverse primer in the neomycin coding sequence. The 3′ external probe (Right Arm) hybridizes to 12.3-kb (*Bcor^+/Y^*) and 10.5-kb (*Bcor^Neo/Y^*) *BglII* fragments. (C) The *Bcor* ^Δ*3*^ allele encodes an N-terminally truncated version of BCOR. The anticipated and actual translation open reading frames for the *Bcor* ^Δ*3*^ allele are diagrammed (top). Western blot analysis on the eyes of *Bcor^+/+^*, *Bcor* ^Δ*3*^/+ and *Bcor* ^Δ*3/Y*^ mice (bottom) reveals the presence of an N-terminally truncated version of BCOR (red arrows) in both the *Bcor* ^Δ*3*^/+ and *Bcor* ^Δ*3/Y*^ lanes, migrating at a slightly lower molecular weight in comparison to wild type BCOR (black arrows).

To investigate the role of the X-linked *Bcor* gene in development, we generated a conditional *Bcor* allele, *Bcor^Fl^*, in which exon 3 is flanked by loxP sites to allow its removal via expression of Cre recombinase (Figure 1A and targeting verification Figure 1B). We hypothesized that excision of exon 3 (*Bcor* ^Δ*3*^) would result in a frame shift and a premature stop codon that should cause severe carboxy-terminal deletion of the BCOR protein and/or elimination of the mRNA by nonsense-mediated decay (Figure 1C). Based on human OFCD patients we expected that heterozygous female mice carrying this deletion in all tissues would recapitulate the phenotype of human OFCD patients and that hemizygous male mice would not be viable. Unexpectedly, breeding *Bcor^Fl/+^* mice to ß-actin-Cre mice generated apparently normal female (*Bcor* ^Δ*3/+*^) and male (*Bcor* ^Δ*3/Y*^) mice. Western blot analysis of BCOR protein from the eyes of these mice and wild type controls revealed that an alternative start codon, 3′ to the engineered frame shift, was used for translation, generating BCOR protein with a predicted 71 amino acid amino-terminal deletion (Figure 1C). Thus, although this amino-terminal region is conserved, with 69% identity to the *Xenopus* protein and 90% identity to that of human, it is not essential.

### Generation and analysis of *Bcor^Neo^* and *Bcor^Gt^* alleles in mice

**Figure 2:**
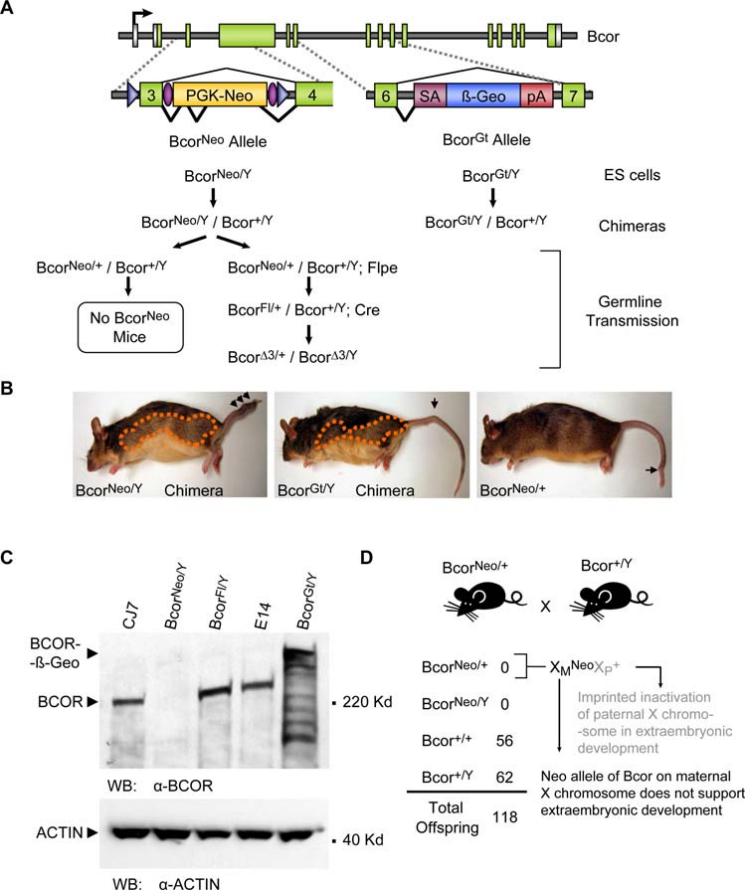
Transmission of the *Bcor^Neo^* and *Bcor^Gt^* alleles in mice. (A) Diagram of *Bcor^Neo^* and *Bcor^Gt^* allele transmission. Location of each genomic alteration is shown, respective to the *Bcor* locus (top). The splicing pattern of each allele is indicated with the more predominant splicing pattern drawn below each allele in a darker line. See Supplementary Figure 1 for detailed analysis. The mating schemes for *Bcor^Neo^* and *Bcor^Gt^* mouse lines are depicted below the genomic alterations. (B) *Bcor^Neo^* and *Bcor^Gt^* chimeric mice and *Bcor^Neo/+^* mice display similar phenotypes. Coat color contribution bias (orange dashed outline) and malformed kinked tail phenotype (black arrows) were present in both *Bcor^Neo^* and *Bcor^Gt^* chimeric mouse lines. *Bcor^Neo/+^* offspring of *Bcor^Neo^* chimeric fathers also displayed the malformed kinked tail phenotype. (C) Western blot analysis of CJ7 (*Bcor^Neo/Y^* parent), *Bcor^Neo/Y^*, *Bcor^Fl/Y^* (rescued line), E14 (*Bcor^Gt/Y^* parent) and *Bcor^Gt/Y^* ES cell lines. The top panel was probed with an anti-BCOR antibody and anti-ACTIN antibody was used as a loading control on the bottom panel. BCOR protein is undetectable in *Bcor^Neo/Y^* ES cells. *Bcor^Fl/Y^* rescued ES cells show wild type levels of BCOR. *Bcor^Gt/Y^* ES cells show a large BCOR-ß-Geo fusion protein with multiple breakdown products. (D) Parent-of-origin effect of *Bcor^Neo^* allele. Matings between *Bcor^Neo/+^* females (offspring of *Bcor^Neo^* male chimeras) and wild type males do not produce any offspring carrying the *Bcor^Neo^* allele. In mice, the paternal X chromosome is selectively inactivated in extraembryonic tissue whereas in the embryo proper X inactivation occurs randomly (grey text indicates paternally imprinted X inactivation). The female lethality observed likely results from paternally imprinted X-inactivation of the wild type *Bcor* allele in extraembryonic tissue.

Although the *Bcor^Fl^* mutation did not provide the expected null allele, during the initial targeting of the *Bcor* locus we fortuitously created a loss of function *Bcor* allele (*Bcor^Neo^*) that provided insights into *Bcor* function both in mice and in ES cell differentiation. We included a Neomycin (*Neo*) selection cassette in the *Bcor* targeting vector. Often the *Neo* cassette is excised during mRNA splicing to generate a normal transcript. However, in some cases a proportion of mRNAs are spliced into and then out of *Neo*, and the insertion of *Neo* sequences can result in a hypomorphic or null allele [23], [24]. Phenotypic effects of such aberrant splicing in chimeric animals would be most readily observed for X-linked genes like *Bcor* since XY ES cells are routinely used for gene targeting. Upon generation of chimeric mice with our *Bcor^Neo/Y^* ES cells (Figure 2A) we observed two prominent phenotypes suggesting that the *Neo* cassette was indeed interfering with *Bcor* expression and function (Figure 2B). First, 3 out of 10 *Bcor^Neo^* chimeras had kinked and sometimes shortened tails, suggesting a possible neural tube defect [25]. Second, 10 out of 10 chimeras exhibited a consistent bias in coat color contribution of the 129 (Agouti) ES cells to the sides of the bodies and legs of the mice. Since Agouti locus expressing cells in the skin are derived from the ectodermal lineage, this indicates a selective bias against *Bcor^Neo/Y^* cells to contribute to certain ectodermal lineages. The same tail and coat color phenotype was also observed in 3 out of 3 chimeric mice that we generated from the Bay Genomics ES cell line, XE541 (*Bcor^Gt^*) (Figure 2B). The XE541 ES cell line harbors an insertional gene trap mutation (ß-Geo) in the 6^th^ intron of mouse *Bcor*, which is predicted to result in an N-terminal BCOR-ß-Geo fusion protein (Figure 2A).

### Molecular analysis of *Bcor^Neo^* and *Bcor^Gt^* alleles

To establish the molecular nature of the expression defects of the *Bcor^Neo^* and *Bcor^Gt^* alleles in ES cells, we examined *Bcor* mRNA splicing patterns by RT-PCR and proteins levels by western blot analysis. The *Bcor^Neo^* allele ES cells had a mixture of transcripts spliced to exclude *Neo* (Figure 2A, left upper splicing pattern and Figure S1A), which encode intact BCOR, together with transcripts spliced to incorporate the *Neo* cassette (Figure 2A, left lower splicing pattern and Figure S1A). Sequencing of RT-PCR products demonstrated that the two regions of the Phosphoglycerate Kinase-Neomycin (Pgk-*Neo*) cassette were recognized as exons by the splicing machinery (Figure S1B). The first exon was 86 nucleotides from the Pgk promoter and the second utilized a splice acceptor site within the 5′ UTR of the *Neo* gene cassette and a donor site within the 3′UTR. This latter exon was identical to that used by other previously reported targeted genes [24]. The result of this splicing is the introduction of a premature in frame stop codon derived from the Pgk promoter sequence. Splicing into *Neo* predominates, as full length BCOR protein levels are not detectable by western blot analysis in the *Bcor^Neo^* allele ES cells as compared to the parental ES cell line CJ7 (Figure 2C). We conclude that the *Bcor^Neo^* allele is a severe loss of function allele but because we did detect some correctly spliced transcript it may not be a null allele.

The transcripts from the *Bcor^G^* ^t^ allele are a mixture of those that splice over the gene trap cassette (Figure 2A, right upper splicing panel and Figure S1B) which encode intact BCOR and those that splice into the gene trap ß-Geo cassette, truncating the gene (Figure 2A, right, lower splicing pattern and Figure S1B). By western blot analysis the large BCOR-ß-Geo fusion protein and multiple breakdown products are observed (Figure 2C). Although a faint band corresponding to full length BCOR is present this may represent a co-migrating BCOR-ß-Geo degradation product. The BCOR-ß-Geo fusion protein lacks the region required for interaction with the PcG proteins [4] and therefore is predicted to lack transcriptional repression activity. Although the fusion protein appears to be unstable, the total amount of protein is significantly higher than the level of intact BCOR found in the parental ES cell line E14 (Figure 2C). Consistent with this we found, using quantitative-RT-PCR, that the *Bcor-ß-Geo* fusion transcript level is 10-fold higher than the level of intact *Bcor* transcript found in the parental ES cell line. This suggests that intact BCOR negatively autoregulates its own transcription while the BCOR-ß-Geo fusion is unable to function in this capacity. In summary, both the *Bcor^Neo^* and *Bcor^Gt^* alleles represent severe loss of function mutations and result in kinked and shortened tails and a coat color bias in chimeric animals.

### Parent-of-origin effect of *Bcor^Neo^* allele

To further characterize the effect of these two loss of function mutations in mice we bred the male chimeras to wild type females to obtain germline transmission of the alleles. This was only successful with the *Bcor^Neo^* allele and we were able to produce *Bcor^Neo/+^* females. (Because *Bcor* is X-linked, *Bcor^Neo/Y^* males cannot be generated from male chimeras.) Germline transmission of the *Bcor^Gt^* allele did not occur and there are at least two possible explanations for this. First, the *Bcor^Gt^* allele may not be compatible with germline development. Second, since only three *Bcor^Gt^* chimeric mice were obtained and bred, it is possible that upon generation of more chimeras germline transmission would occur. We bred the female *Bcor^Neo/+^* mice extensively but, in contrast to the chimeric males, they never produced any *Bcor^Neo/+^* offspring (Figure 2A and D). (*Bcor^Neo/Y^* males, based on the lack of male OFCD patients, are predicted to die during embryonic development.) However, we were able to breed the female *Bcor^Neo/+^* mice to FLPe recombinase expressing mice to remove *Neo*, and got viable *Bcor^Fl/+^* and *Bcor^Fl/Y^* progeny (Figure 2A). In mice, X chromosome inactivation differs between the embryo and the extraembryonic tissue. The embryo undergoes random X-inactivation, in contrast to the extraembryonic tissue where the paternally-derived X chromosome is inactivated [26]. The female lethality observed, arising when the mutant X-linked allele comes from the mother, likely results from paternally-imprinted X-inactivation of the wild type *Bcor* allele in extra-embryonic tissue of the female offspring (Figure 2D and see discussion). Thus, our results are consistent with a requirement for *Bcor* in extraembryonic tissue where *Bcor* is normally highly expressed [22]. The practical consequence of this parent-of-origin effect was that we could not generate extensive numbers of *Bcor^Neo/+^* mice and thus phenotypic analysis was limited to the *Bcor^Neo^* and *Bcor^Gt^* allele-containing chimeras and the remaining six aging female *Bcor^Neo/+^* mice that were generated from the two germline-transmitting male chimeras we obtained.

### Effect of *Bcor^Neo^* allele on eye development

In OFCD congenital cataracts have been described in all patients. We examined the eyes of the remaining *Bcor^Neo/+^* mice when they ranged in age from 7 to 22 months. Using a slit lamp, the mice were examined for lens opacification, which is indicative of catararacts. Eight out of twelve eyes (67%) in the six *Bcor^Neo/+^* mice displayed lens opacification. Although we do not know the age of onset, two of *Bcor^Neo/+^* mice that were 7 months of age had bilateral lens opacification. We did not have aged matched C57Bl/6∶129 mixed background control animals. However, the frequency of cataracts in the *Bcor^Neo/+^* mice was substantially higher than that reported for either C57Bl/6 or 129 inbred stains (C57Bl/6 is 25% at 14 months [27] and 129 0% at 13–17 months, as detected by slit lamp analysis [28]). Over seventy percent of OFCD patients have microphthalmia. By visual inspection we only observed one *Bcor^Neo^* allele-containing chimera with obvious microphthalmia. However, we did not perform postmortem globe measurements so we cannot rule out the possibility that additional mice had more subtle microphthalmia.

### Analysis of *Bcor^Neo/Y^* and *Bcor^Gt/Y^* cell contribution to hematopoietic development

In OFCD patients 90–100% of surviving white blood cells show inactivation of the X chromosome carrying the mutant allele of *Bcor*, indicating that cells expressing the mutant allele of *Bcor* are at a selective disadvantage [18]. Because the majority of white blood cells are neutrophils and T cells (and to a lesser extent B cells) this strongly suggests that *Bcor* plays an important role in the development of these hematopoietic lineages.

**Figure 3:**
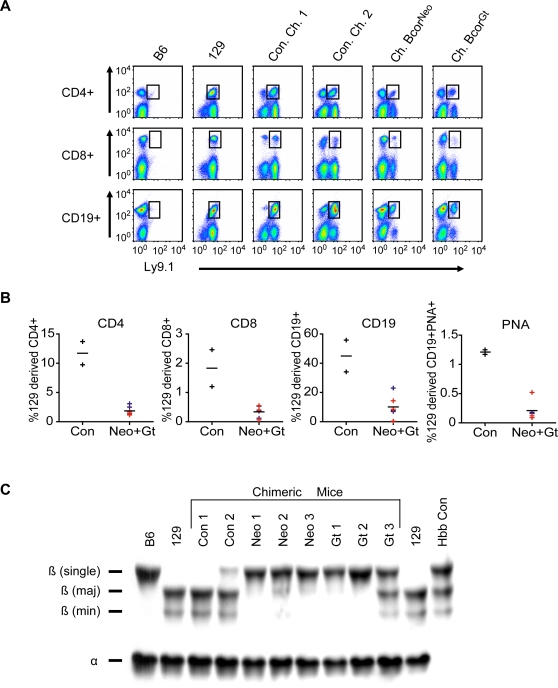
Analyses of *Bcor^Neo/Y^* and *Bcor^Gt/Y^* cell contribution to hematopoietic development in chimeric mice. (A) The *Bcor^Neo/Y^* and *Bcor^Gt/Y^* 129 strain derived ES cells can contribute to the B and T lineages. White blood cells from the spleens of *Bcor^Neo/Y^* and *Bcor^Gt/Y^* and appropriate controls were stained with antibodies to the 129 strain specific surface antigen Ly9.1 and the T cell markers CD4 and CD8 and B cell marker CD19. Cells were analyzed by flow cytometry and results are displayed as density plots with black boxes gating T or B cell populations of 129 origin. B6 and 129 strain wild type mice control for Ly9.1 specificity. CJ7 and E14 129 mouse strain derived ES cells were used to generate control chimera 1 (Con. Ch. 1) and control chimera 2 (Con. Ch. 2). Flow cytometry analyses were performed on chimeric control mice and on three mice for each mutant chimeric line, of which an example scatter plot is displayed for each. (B) Compared to chimeric controls, *Bcor^Neo/Y^* and *Bcor^Gt/Y^* 129 strain derived ES cell contribution to B and T cells is reduced. Graphs of scatter plot data show 129 strain contributions to each hematopoietic lineage. Black bars indicate the average contribution of control chimeric mice 1 and 2 (Con, black +) and the combined average of three *Bcor^Neo^* (Neo, red +) and three *Bcor^Gt^* (Gt, blue +) mice. Contribution to germinal center B cells is also reduced compared to controls as indicated by the final plot showing the PNA+ (peanut agglutinin; germinal center marker) percentage of CD19+ 129 derived B cells. P values were ≤0.05 for all hematopoietic cell populations tested (CD4; 0.004, CD8; 0.034, CD19; 0.023 and PNA; 0.001). (C) *Bcor^Neo/Y^* and *Bcor^Gt/Y^* ES cells can contribute to adult erythrocytes in chimeric mice but contribution is reduced in comparison to controls. Coomassie stained gel of hemolysates from erythrocytes of mice described above in panels A and B. *Bcor^Neo^* and *Bcor^Gt^* chimeric mice (Neo 1, 2 and 3 and Gt 1, 2 and 3) primarily express B6 derived ß -single globin where as chimeric control mice 1 and 2 primarily express 129 derived ß -major and ß -minor globin. The hemoglobin control lane (Hbb Con) was prepared from samples that contain ß -single, ß -major and ß -minor globin.

We hypothesized that the loss of function mutations in the *Bcor^Neo/Y^* and *Bcor^Gt/Y^* ES cell would result in reduced or blocked contribution to hematopoietic lineages in our chimeric mice. The contributions of *Bcor^Neo/Y^* and *Bcor^Gt/Y^* 129 strain ES cells to splenic B and T cells of 129/B6 chimeric animals were analyzed by fluorescence activated cell sorting (FACS). We used the 129 strain-specific B and T cell surface antigen Ly9.1 [29], [30], the commonly used T-cell markers CD4 and CD8, and the B-cell marker CD19. We found that Ly9.1 positive cells can contribute to CD4, CD8 and C19 positive hematopoietic populations in *Bcor^Neo/Y^* and *Bcor^Gt/Y^* chimeric mice (Figure 3A and B). The 129 contribution to each hematopoietic cell type tested was reduced relative to two control chimeras in three *Bcor^Neo/Y^* and three *Bcor^Gt/Y^* chimeric mice. CD4+, CD8+ and CD19+ were reduced compared to control chimeras on average by 87%, 86% and 85%, respectively. Thus, the *Bcor^Neo/Y^* and *Bcor^Gt/Y^* ES cells can contribute to the B and T lineages but they appear to be at a selective disadvantage, as in OFCD patients, suggesting that *Bcor* is important for murine B and T cell development.

Because BCOR is a known corepressor of BCL6, an important regulator of germinal center formation in B-cell maturation, we hypothesized that *Bcor^Neo/Y^* and *Bcor^Gt/Y^* cell contribution to germinal center formation might be blocked or reduced in chimeras. To test this, we looked for the presence of PNA, a germinal center marker, in the CD19+ and Ly9.1+ cell population in cells harvested from the spleens of both mutant chimeric lines. Similar to the B and T cell analysis, we observed an 87% reduction compared to control chimeras in *Bcor^Neo/Y^* and *Bcor^Gt/Y^* contribution to CD19+ PNA+ cells suggesting a compromised germinal center reaction (Figure 3B). To assess the ability of *Bcor^Gt/Y^* and *Bcor^Neo/Y^* ES cells to contribute to adult erythrocyte lineages in chimeras, a hemoglobin analysis was performed, relying on genotype differences between host and ES-derived cells. Erythrocytes derived from the 129 genetic background express ß-major and ß-minor globin, while B6 erythrocytes express ß-single globin [31], [32]. We found that *Bcor^Neo/Y^* and *Bcor^Gt/Y^* ES cells can contribute to mature red blood cells but the contribution is severely reduced compared to the controls (Figure 3C).

### Analysis of *in vitro* differentiation potential of *Bcor^Neo/Y^* and *Bcor^Gt/Y^* ES cells

The absence of OFCD males indicates an early and essential role for *Bcor* in human development. The parent-of-origin effect strongly suggests that *Bcor* plays an essential role in extraembryonic tissue but *Bcor* may play additional roles earlier in development. We used our two loss of function ES cell lines in *in vitro* differentiation assays to test whether *Bcor* plays a role in ES cell differentiation. ES cells cultured in suspension in the absence of leukemia inhibitory factor (LIF) undergo primary differentiation after 2 to 4 days to form simple embryoid bodies (EBs) [33], [34]. These contain an outer endoderm layer surrounding an inner cell mass. Around day 4, differentiation of columnar epithelium with a basal lamina and formation of a central cavity occurs. Such cystic EBs bear similarities to the egg cylinder-stage embryos [35]–[37]. By 6 days of differentiation, EBs are comparable to early organogenesis-stage embryos (E7.5) [38]. EB cells can be dispersed and induced with appropriate growth conditions to undergo secondary differentiation into particular cell lineages [34], [39].

**Figure 4:**
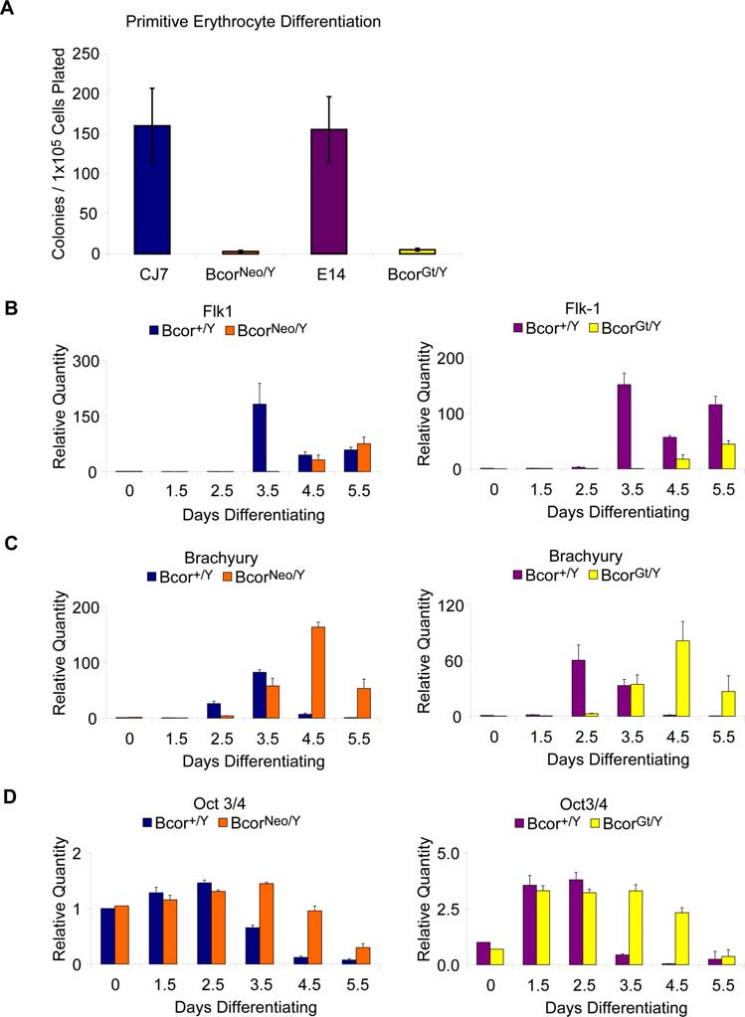
*In vitro* differentiation of *Bcor^Neo/Y^* and *Bcor^Gt/Y^* ES cell lines. (A) *Bcor^Neo/Y^* and *Bcor^Gt/Y^* ES cell lines display a reduced ability to form primitive erythrocyte colonies in comparison to control CJ7 and E14 parental lines. ES cell were differentiated into embryoid bodies for 4.5 days, dispersed and replated in methylcellulose primitive erythrocyte colony forming assays. Error bars represent the standard error of the mean for each ES cell line differentiated (CJ7 and *Bcor^Neo/Y^*; n = 3, E14 and *Bcor^Gt/Y^*; n = 5). (B–D) qRT-PCR analyses of gene expression were performed on embryoid body differentiations (0–5.5 days) of *Bcor^Neo/Y^* and *Bcor^Gt/Y^* ES cell lines and respective parental control lines CJ7 and E14 (*Bcor^+/Y^*). Differentiations were performed in triplicate excluding the day 0 time point. Error bars represent the standard deviation within each time point of the samples analyzed. (B) The expression of hematopoietic lineage marker, *Flk1*, is delayed approximately 1 day in *Bcor^Neo/Y^* and *Bcor^Gt/Y^* differentiating embryoid bodies. (C) The expression of mesoderm and early hematopoietic lineage marker *Brachyury* is similarly delayed 1–2 days in *Bcor^Neo/Y^* and *Bcor^Gt/Y^* differentiating embryoid bodies. (D) The repression of pluripotency marker, *Oct3/4*, is delayed 1–2 days in *Bcor^Neo/Y^* and *Bcor^Gt/Y^* differentiating embryoid bodies.

Both the severe skewing of X-inactivation seen in OFCD patients and the limited contribution of *Bcor* mutant ES cells to hematopoietic lineages in our chimeric animals indicate that *Bcor* plays a role in hematopoiesis. Primitive erythrocytes are the earliest hematopoietic cell types to form *in vivo* [34], [40]. Therefore, to determine whether *Bcor* plays an early role in hematopoiesis, we first examined the ability of dispersed embryoid bodies from our *Bcor^Neo/Y^* and *Bcor^Gt/Y^* ES cell lines to differentiate into primitive erythrocyte (EryP) colonies in the presence of appropriate factors [41], [42]. We found EryP colony formation with CJ7 derived *Bcor^Neo/Y^* and E14 derived *Bcor^Gt/Y^* cell lines is reduced relative to the matched parental ES cell lines (98% and 97% respectively, Figure 4A).

We next used quantitative real-time polymerase chain reaction (qRT-PCR) to examine expression of Flk1 (KDR), an early marker of hematopoietic differentiation potential that is required for primitive and definitive hematopoiesis [43], [44]. RNA samples were collected from ES cells that were allowed to differentiate into embryoid bodies for 0 to 5.5 days. We found that the induction of Flk1 expression in *Bcor^Neo/Y^* and *Bcor^Gt/Y^* cell lines is delayed by one day relative to the parental cell lines and in the time course of the experiment Flk1 expression only reaches ∼40% of the peak expression seen in the parental cell lines (Figure 4B). Moving back in developmental time, we examined expression of Brachyury (T), a marker for the entire mesodermal lineage which in combination with Flk1 marks the hemangioblast cell population [43]. We saw a delay in expression of about 1–2 days but expression does eventually peak at levels comparable to the parental ES cell lines (Figure 4C). Finally, knowing that *Bcor* is expressed in ES cells, we examined the expression of Oct3/4 (Pou5f1), a marker of ES cell pluripotency [45], [46], and found a one to two day delay in repression of Oct3/4 (Figure 4D).

**Figure 5:**
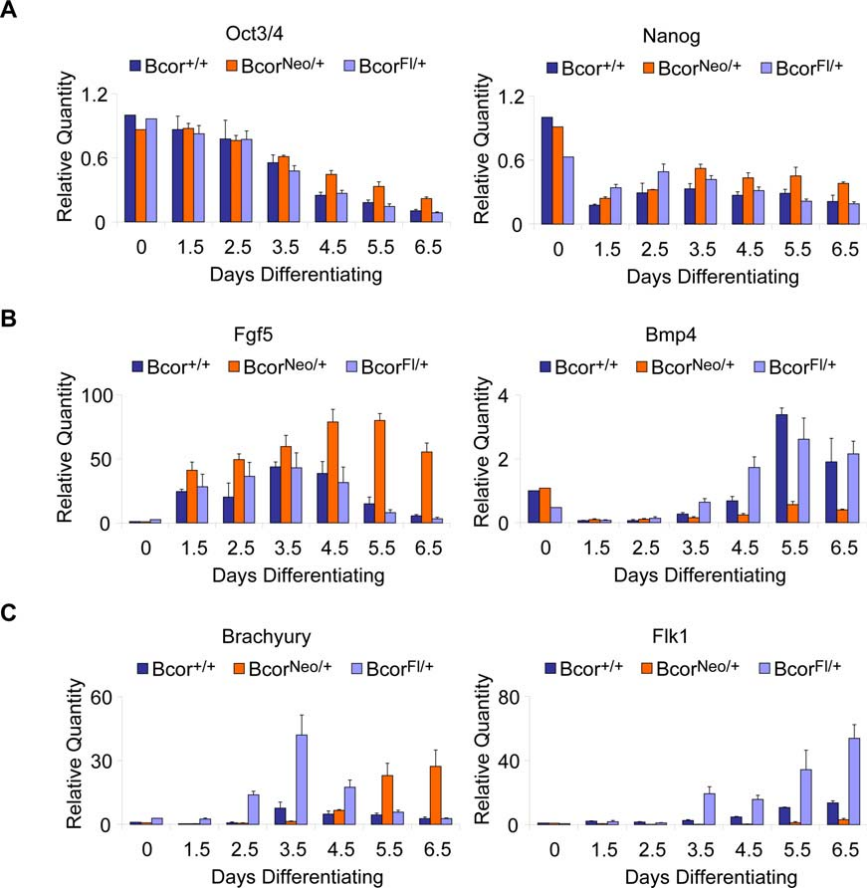
*In vitro* differentiation of *Bcor^Fl/Y^* “rescued” ES cell lines. (A–C) *Bcor^Fl/Y^* “rescued” ES cell lines were created through removal of the *Neo* selection cassette via Flpo recombinase *in vitro*. *Bcor^Fl/Y^* ES cells were clonally selected, screened for *Neo* removal and assessed for rescue of BCOR protein expression by western blot (Figure 2C). qRT-PCR analyses of gene expression during embryoid body differentiation (0–6.5 days) of *Bcor^+/Y^* (CJ7, wild type parent line), *Bcor^Neo/Y^* and three independent *Bcor^Fl/Y^* “rescued” ES cell lines. Differentiations were performed in triplicate excluding the day 0 time point. *Bcor^Fl/Y^* results are the average of 3 independent *Bcor^Fl/Y^* “rescued” lines each differentiated in triplicate. (A) The repression of pluripotency markers, *Oct3/4* and *Nanog*, is restored in *Bcor^Fl/Y^* differentiating embryoid bodies in comparison to *Bcor^Neo/Y^*. (B) Repression of the primitive ectoderm marker, *Fgf5*, and expression of extraembryonic ectoderm marker, *Bmp4*, is restored in *Bcor^Fl/Y^* differentiating embryoid bodies. (C) Timing of expression of the early mesoderm and hematopoietic lineage markers, *Brachyury* and *Flk1*, is restored in *Bcor^Fl/Y^* differentiating embryoid bodies; however expression levels are restored to a higher magnitude with respect to *Bcor^+/Y^* controls (CJ7, wild type parent line).

To confirm that these effects on ES cell differentiation are the result of the *Neo* insertion in *Bcor*, we removed the *Neo* cassette from the *Bcor^Neo^* ES cell line. We generated three independent clonal *Bcor^Fl/Y^* ES cell lines via expression of Flpo recombinase [47] in *Bcor^Neo^* ES cells. We found that these three *Bcor^Fl/Y^* “rescued” lines restore the timing of expression of various ES cell and differentiation markers to that of the parental wild type ES cells (Figure 5) demonstrating that the effects on differentiation were due to the interruption of *Bcor* by the *Neo* cassette. Included in the analysis were a second ES cell marker, *Nanog*, the primitive ectoderm marker, *Fgf5*, [48], [49] and the extraembryonic ectoderm marker, *Bmp4* [35], [50], [51]. In the *Bcor^Neo^* cells we observed a delay in the downregulation of the ES cell markers *Oct3/4* and *Nanog*, whereas *Fgf5* was still up regulated appropriately but its down regulation was delayed by two days. Subsequently, activation of *Bmp4*, *Brachyury* and *Flk1* were all delayed by one to two days. For the genes that are normally expressed earlier in differentiation, the rescued lines restore both the timing and the level of gene expression to that of the parental cell line. However with *Brachyury* and *Flk1*, even though the normal timing of gene expression was restored, inexplicably the level was increased beyond the parental line. We also compared the proliferation rates and cell cycle profile of the parental CJ7 ES cell line with the *Bcor^Neo/Y^* and the three rescued *Bcor^Fl/Y^* ES cell lines and detected no significant differences (data not shown).

Together these ES cell differentiation and expression studies indicate that *Bcor* plays a role in the regulation of gene expression very early in ES cell differentiation and in the mesodermal and ectodermal lineages.

## Discussion

In this study we have used a combination of mouse molecular genetics and ES cell differentiation to identify roles of *Bcor* in early development. These studies provide insights into the possible causes of male embryonic lethality in OFCD, the hematopoietic phenotype of female OFCD patients and the parent-of-origin effect in mice. We have two major findings, as discussed below.

First, we found that in mice the mutant *Bcor^Neo^* allele exhibits a parent-of-origin effect, which together with the high level of *Bcor* expression in extraembryonic tissue [22] strongly suggest an essential requirement for *Bcor* in extraembryonic tissue. The X chromosome has been implicated in causing several malformations of the placenta [52] and our work suggests that *Bcor* is likely to be one of the genes responsible.

There are two alternative but less likely explanations for the parent-of-origin effect that we have considered. First, it is possible that *Bcor* is required very early in development (beginning as early as the 4 to 8 cell stage) when the whole embryo is subject paternal X chromosome inactivation. Once the inner cell mass has formed the cells of the embryo proper are subject to random X inactivation. Paternal X-inactivation is maintained and becomes more complete in the trophectoderm and primitive endoderm, which go on to form to the extraembryonic tissue. However, given the incomplete nature of the paternal imprinting during the 4 cell to blastocyst stage it is most likely that sufficient levels of wild type *Bcor* are transcribed to support development.

A second, albeit unlikely, alternative explanation for the parent-of-origin effect observed in the mouse is that *Bcor* is imprinted such that only the maternal allele is expressed in the embryo proper. However, if such imprinting [53]–[55] occurred in humans, the mother to daughter transmission seen in OFCD patients [56], [57] could not take place since the daughter would only express the mutant *Bcor* allele and would be equivalent to the inviable hemizygous males.

Our second major finding was that in ES cell differentiation studies *Bcor* is required for formation of primitive erythrocytes and proper expression of genes that regulate ES cell pluripotency and genes that drive ectodermal and mesodermal development. These defects together with the apparent limited contribution of *Bcor^Neo/Y^* and *Bcor^Gt/Y^* cells to B and T cells and adult erythrocytes may provide an explanation for the severe skewing of X-inactivation seen in peripheral white blood cells of OFCD patients and indicate a critical early role for *Bcor* in helping to establish hematopoietic development. In data not included, we do not see significant changes in expression of the few endoderm specific genes we tested. Since the number of genes tested was not exhaustive, Bcor may be affecting the expression of other genes important for endoderm development that are yet unidentified.

One of the genes that we found to be misregulated in *Bcor^Neo/Y^* and *Bcor^Gt/Y^* ES cell differentiation assays was *Brachyury*. Mice heterozygous for the *Brachyury* T mutation exhibit a variable short-tailed phenotype [58] while other mutations such as *Brachyury* T^137^ have a kinked or bent tail [59]. Thus mis-expression of *Brachyury* is a possible explanation for the kinked and shortened tails we observed in *Bcor^Neo/Y^* and *Bcor^Gt/Y^* chimeras. The extraembryonic ectoderm marker, *Bmp4*, was also misregulated in *Bcor^Neo/Y^* ES cell differentiation assays. *Bmp4* knock out mice do not express *Brachyury* and display impaired mesoderm differentiation, including reduced extraembryonic mesoderm blood island formation and disorganized posterior structures, further implicating *Bcor* in mesoderm development [49].

Polycomb group proteins (PcG) have been shown to play a central role in stem cell maintenance and lineage specification [60]. The BCOR complex contains several PcG proteins, including RNF2, an H2A ubiquitin ligase, and NSPC1, a BMI1 homolog [4]. RNF2 has been shown to be present at over 1,200 targets in ES cells [61]. At many genes, the PcG complex, PRC2, serves to recruit RNF2 containing complexes. However, about one quarter of ES cell RNF2 targets are not co-occupied with PRC2 components, suggesting RNF2 can be recruited via other means. We hypothesize that BCOR, via interaction with site specific transcription factors, can recruit RNF2 and other BCOR complex components to regulate expression of genes in ES cells and their differentiating progeny. Recently, NSPC1 was identified as a protein that when over-expressed can partially rescue the phenotypic properties of undifferentiated mouse ES cells under differentiation-inducing conditions [62]. Over-expression of NSPC1 may be sequestering or diluting the activity of the Bcor complex thereby preventing full transcriptional repression of target genes. This, together with our data, suggests that inappropriate expression of BCOR complex components affects ES cell differentiation.

Our studies suggest two possible causes of male embryonic lethality in OFCD. First, inappropriate regulation of key developmental genes in *Bcor^−/Y^* embryos may cause lethality before implantation. Second, male embryonic lethality may be due to improper development of extraembryonic derived tissue, potentially resulting in placental failure or incomplete chorioallantoic fusion. Conditional inactivation of Bcor in a spatial and temporal manner will be critical in future experiments in order to bypass the strict requirement for Bcor function in early development.

## Materials and Methods

### Animals

All experimental protocols involving mice described in this publication have been approved by the University of Minnesota Institutional Animal Care and Use Committee. The mice were not kept in specific pathogen free (SPF) rodent housing, thus they were exposed to multiple pathogens that are present at sub clinical levels in conventional rodent housing. Due to continual pathogen exposure, PNA+ CD19+ germinal center positive cell populations were already present in our mice thus, we did not specifically induce germinal center reactions through immunization.

### Generation of *Bcor* mutant mice

A mouse *Bcor* cDNA fragment containing sequences from within exon 4 to within exon 7 was used to screen the RPCI-22 129S6/Sv EvTac Bac library (Stratagene) [63], and positive BAC clones were used to clone *Bcor* genomic sequence. The left and right homology arms were cloned into the backbone vector pDZ157 [64]. The final targeting construct is diagrammed in Figure 1A. The *Bcor* targeting vector was linearized with *PmeI* and electroporated into CJ7 ES cells (originally derived from the 129S1 strain). One homologous recombinant was identified from 768 G418-resistant colonies by Southern hybridization using a DNA probe from sequences upstream of the targeting vector to screen genomic DNA digested with *BamH1*. Homologous recombination was confirmed on both ends of the targeted region by Southern hybridization. Probes for Southern hybridization were generated from a BAC clone containing *Bcor* genomic sequence by PCR using primers VBp525/VBp526 (5′ probe, left arm) and VBp582/ VBp583 (3′ probe, right arm). The targeted ES cell clone containing the *Bcor^Neo^* allele was injected into C57Bl/6 blastocysts to generate chimeras. Chimeric males were bred with C57Bl/6 females to generate heterozygotes carrying the *Bcor^Neo^* allele. *Bcor^Neo^* ^/+^ females were bred with male β-*actin-Flpe* transgenic mice to delete the frted sequence and generate heterozygotes carrying the *Bcor^Fl^* allele. *Bcor^Fl/+^* females were bred with male β-*actin-Cre* transgenic mice to delete the floxed sequence and generate heterozygous *Bcor^Δ3/+^* and hemizygous *Bcor^Δ3/Y^* deletion mutants.

VBp525 5′-CTCTACTTGCTCAGTCTGCCTGCAATG-3′

VBp526 5′-AAGTCGACACATTTCCTTTGTTAGCAG-3′

VBp582 5′-GAGTTGTATCTCATAAATTGTGGTTG-3′

VBp583 5′-CTGTCATTCACTTTGAGCCTGGTGT-3′

The XE541 ES cell line was created by the BayGenomics genetrap consortium through insertional genetrap mutagenesis of the wild type E14 ES cell line (originally derived from the 129P2 strain). The genetrap mutation resides in the sixth intron of the *Bcor* gene and results in a loss of function allele we named *Bcor^Gt^*. XE541 ES cells harboring the *Bcor^Gt^* allele were injected into C57Bl/6 blastocysts by the University of Minnesota Mouse Genetics Laboratory to generate chimeric mice.

### Genotyping

The *Bcor^+^*, *Bcor^Fl^* and *Bcor* ^Δ*3*^ alleles were detected by polymerase chain reaction (PCR) from tail clip genomic DNA using primer set VBp573 /VBp981. The described PCR generates an 1122 base pair (bp) amplicon for the *Bcor^Fl^*, a 993 bp amplicon for the *Bcor^+^* allele and a 332 bp amplicon for the *Bcor* ^Δ*3*^. The Bcor^Neo^ allele was detected by separate PCR reaction using primer set, VBp1023/VBp981 and generates a 693 bp amplicon.

VBp573 5′-GCCTGAAGTAGCTGACATGTCTCTGAT-3′

VBp981 5′-AAAGCCCTAGGAACTACTTGGAGGC-3′

VBp1023 5′-CTATCGCCTTCTTGACGAGTTCTTC-3′

### Western Blot Analyses

In both western blot analyses performed, mouse eyes or ES cells were incubated in lysis buffer (1× phosphate buffered saline (PBS), 10% glycerol, 0.5% Nonidet-P40, 2 mM dithiothreitol (DTT), 0.2 mM phenylmethanesulphonylfluoride (PMSF) and 1× Complete Protease Inhibitor (Roche)), sonicated, normalized by Bradford Assay and resolved on a NuPAGE 3–8% Tris-Acetate gel (Invitrogen). Proteins were transferred overnight at 4°C to a nitrocellulose membrane, blocked with non-fat dry milk and incubated with polyclonal anti-BCOR [4] and monoclonal anti-B-Actin antibodies (Abcam 8226).

### Generation of “Rescued” *Bcor^Fl/+^* Embryonic Stem Cells

To remove the *Neo* selection cassette and restore BCOR expression, *Bcor^Neo^* ^/Y^ ES cells were transfected with a mouse codon-optimized FLPo site specific recombinase as described by Raymond and Soriano [47]. Five clonally selected rescued lines in which the *Neo* cassette had been deleted were identified by PCR screening of gDNA with primer sets VBp573 /VBp981 and VBp1023/VBp981.

### Flow Cytometry and Hemoglobin Analyses

White blood cells were harvested into Hank's buffered saline solution (HBSS) at 4°C from the spleens of sacrificed *Bcor^Neo^*, *Bcor^Gt^* and appropriate control mice. Cells were pelleted at 300×g at 4°C for this step and all subsequent centrifugation steps. Red blood cells were lysed and white blood cells were then pelleted and resuspended in a 1∶3 dilution of clone 2.4G2 hybridoma (ATCC HB-197) supernatant for 10 minutes at room temperature to block Fc receptors. Cells were then counted and 2×10^6^ cells were respuspended in 200 uL of FACs buffer (PBS, 1% FCS, and 0.02% azide, pH 7.2) in a 96 well round bottom plate for antibody staining. Cells were stained with a 1∶200 dilution of appropriate primary antibody or lectin (Anti-Ly9.1-FITC (BD Pharmingen), Anti-CD19-PE (eBioscience), Anti-CD4-PerCP (BD Pharmingen), Anti-CD8-APC (eBioscience) and PNA-biotin (BD Pharmingen)) in FACs buffer for 20 minutes at room temperature and washed 1 time with 200 uL of FACs buffer. Cells incubated with PNA-biotin were then incubated with streptavidin-APC for 20 minutes at room temperature. All samples were then washed another 2 times with FACs buffer, fixed in 1% paraformaldehyde and stored at 4°C until flow cytometry was performed on the following day. Cell events were recorded on a flow cytometer (FACSCalibur; BD Biosciences) and analyzed with FlowJo software (Tree Star Inc.). Clarified hemolysates prepared from the erythrocytes of the same mice were resolved by Triton-acid-urea gel electrophoresis and visualized by Coomassie blue staining [32], [65]

### ES Cell Culture and Differentiation

ES cells were maintained in 37°C 5% CO2 incubator on irradiated mouse embryonic fibroblasts (MEFs) in Knock Out Dulbecco's Modified Eagles Medium (KO-DMEM, Invitrogen) supplemented with (15% ES cell certified fetal bovine serum (ES-FBS, Hyclone), 0.1 mM non-essential amino acids (NEAA), 2 mM L-glutamine, 0.1 mM 2-mercaptoethanol (BME), 100 U/ml penicillin, 100 µg/ml streptomycin (Invitrogen) and 1000 U/mL ESGRO (Chemicon)). Protocols for differentiation of ES cells were essentially as described by Zhang et. al. [41], [42] with minor modifications. Briefly, ES cells were passaged one time onto 0.1% gelatinized tissue culture plates to remove MEFs. After 2 days, cells were trypsinized for 5 minutes to a single cell suspension, stopped with fetal bovine serum, counted and plated at low density (5–10×10^4^ cells/mL depending on the experiment) in embryoid body (EB) differentiation media (Iscove's Modified Dulbecco's Medium (IMDM) supplemented with 15% fetal bovine serum (Atlas Biologicals), 50 ug/mL Ascorbic Acid (Sigma), 2 mM L-glutamine, 4.5×10^−4^ M monothioglycerol (MTG) in biological triplicate for each sample on ultra low attachment tissue culture plates (Corning)). EBs were maintained at 37°C 5% CO2 for duration of indicated experiments. Primitive erythrocyte colony forming assays utilized EBs, as described above, incubated until day 4.5. EBs were then trypsinized to a single cells suspension, stopped with FBS, counted and replated at 5×10^3^ cells/mL in triplicate in primitive erythrocyte differentiation media containing (IMDM, 1% methylcellulose (Sigma), 10% plasma derived serum (Animal Technologies Inc.), 12.5 ug/mL Ascorbic Acid (Sigma), 2 mM L-glutamine (Invitrogen), 200 ug/mL transferrin (Roche), 4.5×10^−4^ M MTG (Sigma), 2 U/mL Erythropoietin (Epogen, Roche) and 5% protein free hybridoma media –II (Invitrogen). On day 4 secondary differentiation plates were counted for the presence of primitive erythrocyte colonies based on morphology and color.

### Gene Expression Analysis

Total RNA was extracted with TRIzol (Invitrogen), normalized by spectroscopy and reverse transcribed using either M-MLV or SuperScript III (Invitrogen) according to the manufacturer's protocol. Real-time quantitative PCR was performed using FastStart SYBR Green Master mix (Roche) on a Stratagene M×3000P (Stratagene). Gene expression was determined using the relative quantitation method [66] and Hprt expression was used to normalize all sample template concentrations. Denaturing curves were performed on all reactions to verify homogeneity of the amplified product. The following gene specific primers were used.

Hprt For 5′-AGCTACTGTAATGATCAGTCAACG-3′

Hprt Rev 5′-AGAGGTCCTTTTCACCAGCA-3′

Oct4 For 5′-GAAGCAGAAGAGGATCACCTTG-3′

Oct4 Rev 5′-TTCTTAAGGCTGAGCTGCAAG-3′

Nanog For 5′-CCTCAGCCTCCAGCAGATGC-3′

Nanog Rev 5′-CCGCTTGCACTTCATCCTTTG-3′

Brachyury For 5′-CTCACCAACAAGCTCAATGG-3′

Brachyury Rev 5′-GGTCTCGGGAAAGCAGTGGC-3′

Flk1 For 5′-CACCTGGCACTCTCCACCTTC-3′

Flk1 Rev 5′-GATTTCATCCCACTACCGAAAG-3′

Fgf5 For 5′-CAAAGTCAATGGCTCCCACGAAG-3′

Fgf5 Rev 5′-CTACAATCCCCTGAGACACAGCAAATA-3′

Bmp4 For 5′-CACTGTGAGGAGTTTCCATCACGAAG-3′

Bmp4 Rev 5′-GGATGCTGCTGAGGTTGAAGAGGA-3′

### Statistical analysis

P values in Figure 3 were determined using a Student's t-test, using the natural log of the percent contribution values, assuming unequal variances.

## Supporting Information

Figure S1 Analysis of splicing pattern in *BcorNeo/Y* and *BcorGt/Y* ES cells. (A) Clone 2B1 ES cells, containing the *BcorNeo/Y* allele, aberrantly splice two portions of the Pgk-Neo coding sequence into the *Bcor* transcript. S1–S4 shows the sequence surrounding the splice junctions as determined by sequencing of reverse transcription PCR products generated by primers A–D. Amplicon AB and CD were sequenced to determine splice junctions S1–S3 and S4, respectively. The predominant splice pattern found is indicated by bolded black splice connector lines. VBp1114 (A) ATGCTTTCTGCAACCCCTCTGTAT, VBpNeoRev (B) TCGGCAGGAGCAAGGTGAGAT, VBpNeoFor (C) CCGGTTCTTTTTGTCAAGACCG, VBp1116 (D) TTGTATCCCAGGCGGTGTTTTG. (B) Clone XE541 ES cells, containing the *BcorGt/Y* allele, predominantly splice from exon 6 of *Bcor* into the splice acceptor of the genetrap cassette. Reverse transcription PCR of XE541 ES cell total RNA using primers F and G, generates the expected amplicon of 529 base pairs. Wild type splicing can also be detected in XE541 ES cells as shown by reverse transcription PCR using primers E and H to generate the expected amplicon of 445 base pairs. The predominant splice pattern found is indicated by bolded black splice connector lines. VBp1616 (E) CGTGCAATGATGCGCTTCTC, VBp1074 (F) AGATTCCAGTCAGCTCAGCCGAGA, VBp1071 (G) ATTCAGGCTGCGCAACTGTTGGG, VBp1617 (H) CTTTGGAGATCCGTCTTCGCTT.
(0.97 MB DOC)
