# Supplementary figures and images for: Role of the Transcriptional Corepressor Bcor in Embryonic Stem Cell Differentiation and Early Embryonic Development
Source: PLoS One. 2008 Jul 30;3(7):e2814. doi: 10.1371/journal.pone.0002814 (PMC2535898; doi:10.1371/journal.pone.0002814)

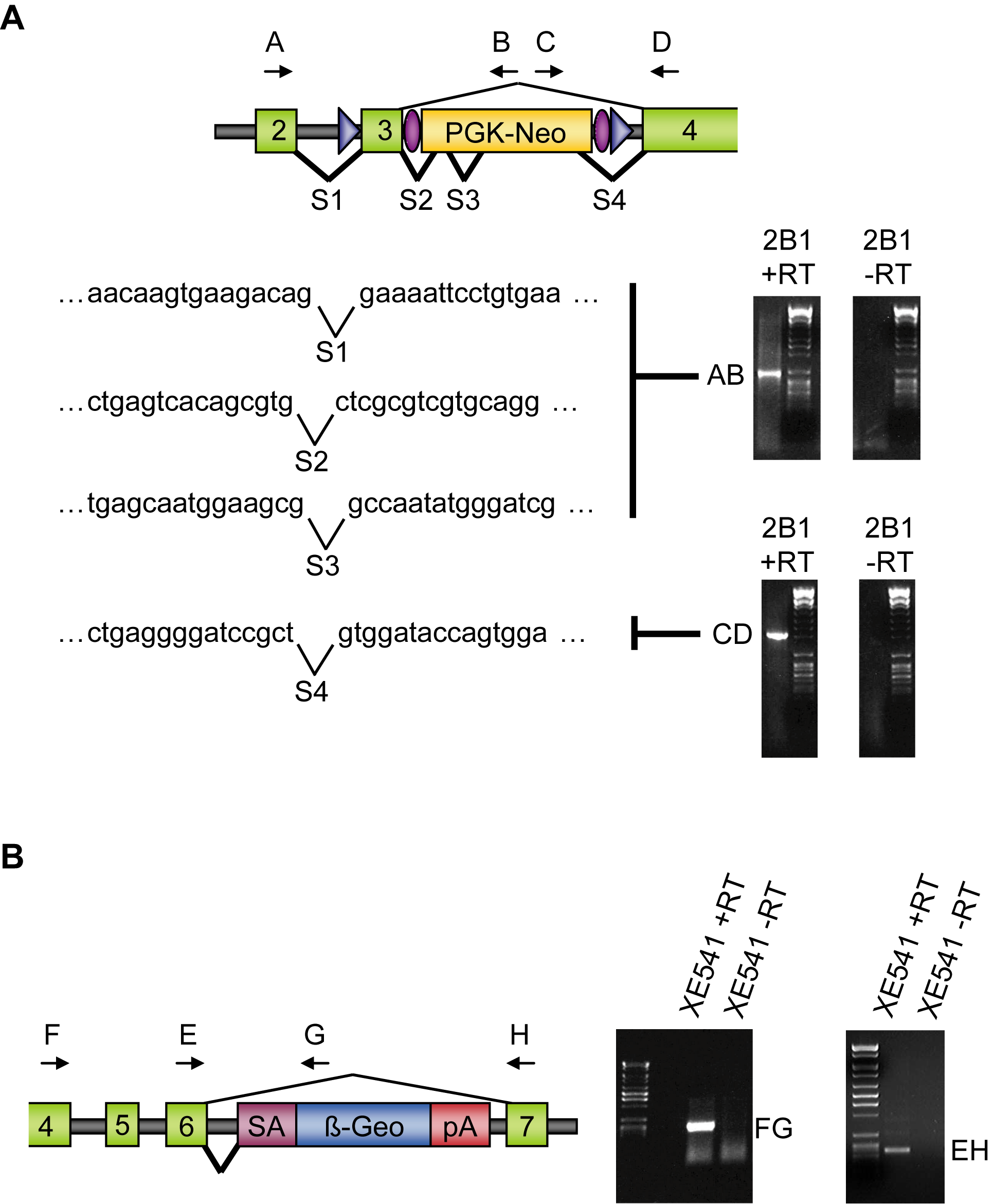

Supplement: Figure S1 — Analysis of splicing pattern in BcorNeo/Y and BcorGt/Y ES cells. (A) Clone 2B1 ES cells, containing the BcorNeo/Y allele, aberrantly splice two portions of the Pgk-Neo coding sequence into the Bcor transcript. S1–S4 shows the sequence surrounding the splice junctions as determined by sequencing of reverse transcription PCR products generated by primers A–D. Amplicon AB and CD were sequenced to determine splice junctions S1–S3 and S4, respectively. The predominant splice pattern found is indicated by bolded black splice connector lines. VBp1114 (A) ATGCTTTCTGCAACCCCTCTGTAT, VBpNeoRev (B) TCGGCAGGAGCAAGGTGAGAT, VBpNeoFor (C) CCGGTTCTTTTTGTCAAGACCG, VBp1116 (D) TTGTATCCCAGGCGGTGTTTTG. (B) Clone XE541 ES cells, containing the BcorGt/Y allele, predominantly splice from exon 6 of Bcor into the splice acceptor of the genetrap cassette. Reverse transcription PCR of XE541 ES cell total RNA using primers F and G, generates the expected amplicon of 529 base pairs. Wild type splicing can also be detected in XE541 ES cells as shown by reverse transcription PCR using primers E and H to generate the expected amplicon of 445 base pairs. The predominant splice pattern found is indicated by bolded black splice connector lines. VBp1616 (E) CGTGCAATGATGCGCTTCTC, VBp1074 (F) AGATTCCAGTCAGCTCAGCCGAGA, VBp1071 (G) ATTCAGGCTGCGCAACTGTTGGG, VBp1617 (H) CTTTGGAGATCCGTCTTCGCTT. (0.97 MB DOC) [file pone.0002814.s001.tif]
